# Commercially produced complementary foods in Bandung City, Indonesia, are often reported to be iron fortified but with less than recommended amounts or suboptimal forms of iron

**DOI:** 10.1111/mcn.12789

**Published:** 2019-06-21

**Authors:** Michele L. Dreyfuss, Mackenzie Green, Agustino, Dian N. Hadihardjono, Doddy Izwardy, Sandra L. Huffman

**Affiliations:** ^1^ Consultant to Helen Keller International New York NY; ^2^ Helen Keller International New York NY; ^3^ Ministry of Health Jakarta Indonesia

**Keywords:** complementary feeding, complementary foods, infant feeding, iron fortification, nutrition labels

## Abstract

Commercially produced complementary foods (CPCF) that are iron fortified can help improve iron status of young children. We conducted a review of 217 CPCF sold in 42 stores in Bandung, Indonesia, in 2017. There were 95 (44%) infant cereals, 71 (33%) snacks or finger foods (biscuits or cookies, puffs, and noodles or crackers), 35 (16%) purees, and 16 (7%) other foods for which we obtained label information. Nearly 70% of CPCF reported iron content on their labels, but only 58% of products were reported to be fortified with iron according to ingredient lists. Among iron‐fortified products, only one fifth indicated a specific type of iron used as the fortificant, but all of these were recommended by the World Health Organization for fortifying complementary foods. Infant cereal was more likely to contain added iron (81%) compared with snacks or finger food (58%) and purees (14%) and had higher iron content per median serving size (cereal = 3.8 mg, snacks or finger food = 1.3 mg, mixed meals = 2.7 mg, and purees = 0.9 mg). Infant cereal was most likely to meet the recommended daily intakes for iron (41% for infants 6–12 months of age and 66% for children 12–36 months) compared with snacks or finger food (infants = 14%, children = 22%), mixed meals (infants = 28%, children = 46%), or purees (infants = 9%, children = 15%). Regulations on fortification of complementary foods need to specify minimum levels and forms of iron and require reporting in relation to requirements by child age and serving size. Monitoring and enforcement of regulations will be essential to ensure compliance.

**Key messages**

- Nearly 70% of 217 commercially produced complementary foods sold in Bandung, Indonesia, contained iron, and 58% were iron fortified. Many of the products contained low or less than recommended amounts of iron.
- Only one fifth of fortified products indicated the type of iron used, and all of these included a form of iron recommended by WHO for fortifying complementary foods.
- Infant cereal was more likely to be fortified and to contain more iron compared with snacks or finger food and purees.
- Complementary food labels should indicate nutrient levels by specific age categories because requirements differ.
- Regulations on fortification of complementary foods should include recommended forms of iron and specify a minimum amount of iron fortification per serving.

## 1 INTRODUCTION

In Indonesia and many parts of Southeast Asia, iron deficiency and anaemia in infants and young children is a public health problem. Approximately one third of young children are anaemic in Indonesia (World Health Organization [WHO], 2015). Petry et al. (2016) estimate that 25% of such anaemia is related to iron deficiency that affects growth and cognitive and motor development (WHO, 2015). Iron intake in young children is often inadequate due to the enhanced iron requirements of infants and young children. Therefore, reducing iron deficiency is a priority of public health programs.

WHO states that fortified complementary foods can play an important role in child feeding (Pan American Health Organization/World Health Organization, 2003) and their role in enhancing child nutritional status has been recognized for decades (Brown & Lutter, 2000; MIYCN [Maternal, Infant and Young Child Nutrition] Working Group‐Subgroup on Formulation Guidelines, 2009). WHO (2010) measures of child feeding include the consumption of iron‐rich foods as an indicator to determine optimal feeding, and fortified complementary foods are often included in the definition of iron‐rich foods. Research by Fahmida, Santika, Kolopaking, and Ferguson (2014) in rural Lombok showed the percentage of infants consuming fortified foods (including infant cereals and biscuits) nearly doubled (42% to 82%) from 2005 to 2010. However, the level and type of iron used in complementary foods has not been studied widely.

Products that state they are iron fortified may have insufficient iron per serving or use poor quality iron fortificants. Codex (amended 2013) revised the standard on fortified complementary foods, and although it does not specify amounts of iron required in such products, it states the “suggested total quantity of … vitamins/minerals contained in a daily ration of the Formulated Complementary Food is at least 50% of the individual nutrient level” that meets the 98th percentile of an individual's nutrition needs. It also states that a serving size of around 50 g is appropriate for infant cereals. Neither the Codex standard for canned baby foods (amended 2017) nor the standard for processed cereal‐based foods for infants and young children (Codex, amended 2017) give recommended iron levels, iron fortificant type, or suggested serving sizes. WHO/FAO states that the following types of iron are suitable for cereal‐based fortified complementary foods: ferrous sulphate, encapsulated ferrous sulphate, ferrous fumarate, or electrolytic iron, all with added vitamin C (Allen, De Benoist, Dary, & Hurrell, 2006). Another effective fortificant is sodium iron EDTA (Hurrell et al., 2010; Yang, Siekmann, & Schofield, 2011). However, there are many less expensive forms of iron often used for fortification with lower effectiveness, including elemental iron and reduced iron (Hurrell et al., 2010).

Codex Alimentarius Commission (2017) standards on canned baby foods states nutrient content should be “expressed in numerical form per 100 g or 100 ml of the food as sold and, where appropriate, as per specified quantity of the food as suggested for consumption.” The Codex standards (amended 2013) state that the total quantity of each vitamin and mineral per feeding of the Formulated Complementary Food ready for consumption is required (Codex Alimentarius Commission, 2013). The Indonesian national regulations (Indonesian Regulation HK 00.06.51.0475/2006 Guideline on Food Label Nutrition Content) state that food products should indicate ingredients, serving sizes, and nutrient content as a percent of the RDA (*Angka Kecukupan Gizi*) per serving. They state that ingredients and specified nutrient levels need to be reported on labels in the Indonesian language if products contain iron greater than 2% recommended dietary allowance (RDA; *Angka Kecukupan Gizi*) per serving, and/or state claims regarding iron content.

The Indonesian DHS reported that 32% of infants and young children 6–23 months of age consumed fortified baby foods (Statistics Indonesia [Badan Pusat Statistik—BPS], National Population and Family Planning Board [BKKBN], and Kementerian Kesehatan [Kemenkes—MOH] & ICF International, 2013). Given the prevalence of iron deficiency anaemia in this population, there is a need to better understand fortification of commercially produced complementary foods (CPCF) in this context. This study assessed labels of CPCF in Indonesia to determine the proportion of products that are fortified at appropriate levels with recommended forms of iron.

## 2 METHODS

This study was conducted as a substudy of an assessment of availability and promotions of CPCF in Bandung City, Indonesia, in 2017 (Green et al., 2019). CPCF were defined as foods recommended for children less than 3 years of age with a label or package that recommends an age of introduction less than 3 years on the label and/or uses the term “baby” or words referring to a child's age to describe the food (e.g., “baby food”), not including infant formula or other breastmilk substitutes (WHO, 2016).

### 2.1 Determination of CPCF sold in Bandung

Master lists of CPCF products for sale in Bandung City were made by reviewing the National Food and Beverage Registry (https://cekbpom.pom.go.id) of the *Badan Pengawasan Obat dan Makanan* (BPOM, National Agency of Food and Drug Control), online research, and informal visits to seven stores in Bandung City that were expected to have a wide variety of CPCF products, based on consultation with local health or nutrition experts. Subsequently, these master lists were used to collect information on which products were sold in 43 stores. Researchers visited 33 purposively selected small stores in closest walking distance to public sector health facilities and 10 large retail outlets purposively selected for their large variety of products following an international protocol (WHO & UNICEF, 2017). CPCF were almost universally available across all 43 stores (97.7%, *n* = 42). Details on store selection can be found in Hadihardjono et al. (2019).

Any additional products found in stores that were not listed in the master list were added. The following criteria were used to include products on the master list:

○ The same product names with different BPOM registry codes were considered one product.
○ The same product names with one or more local manufacturers and one or more international manufacturers (i.e., imported) were considered different products.
○ Single serving and multiserving packages of the same product were considered one product.
○ Different sizes of multiserving packages were considered one product.
○ Bundles of single‐serving sachets or packages were considered a single serving product.
○ Products with the same name with different types of packaging were considered a single product.
○ Different flavours of the same product were considered different products (because their nutrient content or promotions could vary).

### 2.2 Collection and coding of labels of CPCF

In the 42 stores where CPCF were sold, 220 CPCF defined as separate products were found to be for sale. One example of each of these CPCF was purchased for a desk review of the label. Three of these products were not purchased and thus not included in this study. Information from labels were then input into Microsoft Excel (version 2013), including brand and sub‐brand of the product, manufacturer, descriptive name, flavour, recommended age of use, type and amount of iron in the product per 100 g and per serving, and percent of RDA. These were reported in Indonesian or other country RDAs based on the country in which a product was manufactured (as shown in Table 3).

### 2.3 Data preparation and analysis

A total of 217 products were included in this analysis. All CPCF were grouped into the following product type categories: cereals, snacks or finger foods, puddings, mixed meals, and purees. Snacks or finger foods were further classified as biscuits or cookies, puffs, and crackers or noodles because of the heterogeneity of product type. Products were reviewed to determine whether they had recommended age of use, serving size information, iron listed as an ingredient, and iron content information as either a specified amount of iron, a percentage of an RDA, or both. A product was considered iron fortified if its ingredient list contained an iron product or a mineral premix. In cases where a product's ingredient list indicated that iron had been added as a fortificant, but no iron content was listed on the label, a product was considered fortified but not included in the calculations of iron content. If no form of iron was shown as an ingredient, then the product was not considered to be fortified because the Indonesian law requires that fortificants be stated as ingredients. However, it was included in analyses of iron content.

#### 2.3.1 Calculation of iron content

For most products, percentage of the RDA was provided on the label, but no details on iron content were provided. When iron content was given but no percentage of the RDA was reported on the label, then the specified iron content was used for the analyses. However, in the products where both percentage of the RDA and iron content were reported on the label, percentage of the RDA was used.

For products that did not have iron content listed but had percentage of the RDA, iron content (milligrams) was calculated by multiplying the reported percentage of the iron RDA contained in one recommended serving (as listed on the product label) by the RDA for the product's recommended age of use. There were four products that reported containing iron but had no recommended age of use on the label, so iron content was not calculated. In some cases (*n* = 15), the recommended age of use from the BPOM master list differed from that shown on the label. If the product's recommended age of use spanned more than one age category for the specified country's RDA (e.g., 6–24 months), then the average of the RDA values from the two age categories was used to calculate iron content. However, if the recommended age of use was 1–5 years only, the RDA for 1–3 years was used because this was within the age range of interest for complementary foods. The specific country RDA used for this calculation was determined by the information reported on the product label. Table 3 lists the RDAs for these countries that were used in the calculations. Using the initial calculation of iron content for each product, additional iron content variables were generated for different serving sizes of CPCF, including iron per 100 g of product and per median serving size calculated for the products in each category. The median serving size within the category was used rather than that stated on the product because of wide variations in product serving sizes.

#### 2.3.2 Percentage of RDA

In order to calculate the mean RDA for each category of products, the iron content variable (per 100 g or per median serving size) was divided by the age‐specific RDA. A value greater than 100% indicates that a product's iron content was greater than the reference RDA for a specific age range. A percentage < 100% indicates the product had less than 100% of the recommended daily iron for a specific age range. The Indonesian (Indonesia Kementerian Kesehatan RI. Direktorat, 2014) and WHO/FAO (WHO/FAO, 2004) nutrient recommendations were used for this standardized comparison. We used 10% bioavailability for WHO/FAO recommended nutrient intakes (RNI) because the Indonesian DHS reported that 68% of children 6–23 months of age consumed iron‐rich animal foods (meat, fish, poultry, and eggs). This percentage was 28.9% for ages 6–8 months, 62.8% at 9–11 months, and 75.3% at 12–17 months (Statistics Indonesia (Badan Pusat Statistik—BPS), National Population and Family Planning Board (BKKBN), and Kementerian Kesehatan (Kemenkes—MOH) & ICF International, 2013).

#### 2.3.3 Data analysis

Data were cleaned and analysed in Stata/IC 15.1 (StataCorp, 2017). The frequency of each category of CPCF was tabulated, and subsequent analyses were stratified by this variable. Proportions and mean ± standard deviation (*SD*) were used to describe the characteristics of CPCF, including recommended age of use and the recommended serving size, and to categorize products by iron content and fortification. Iron content per 100 g of product and per median serving size were reported as means ± *SD*, and the percentage of the RDA met by the iron content of CPCF was reported as a proportion with 95% confidence intervals. The reported iron content of CPCF was compared between iron‐fortified and unfortified products. All three outcome variables were disaggregated by CPCF product type to compare iron content and adequacy across these groups.

## 3 RESULTS

There were 217 CPCF labels reviewed, including infant cereal (44%, *n* = 95), snacks or finger food (33%, *n* = 71), pureed food (16%, *n* = 35), pudding (3%, *n* = 7), mixed meals (1%, *n* = 3), and other various foods (3%, *n* = 6). Snacks or finger foods comprised a variety of products that were classified as biscuits or cookies (55%, *n* = 39), puffs (24%, *n* = 17), and noodles or crackers (21%, *n* = 15). The recommended age of use on product labels varied by the type of product; most cereals were recommended for use beginning at 6 months of age (Table 1). By contrast, approximately 60% of snacks or finger food, 70% of pureed food, and 100% of mixed meals and pudding were recommended for use starting at 1 year of age. The serving size on the product labels of cereal, mixed meals, and pureed food was somewhat homogeneous for these product types but varied greatly for snacks or finger food, the median ranging from 7.0 g (interquartile range [IQR]: 7.0 g) for puffs to 21.8 g (IQR: 19.6–28.0) for biscuits or cookies to 50.0 g (IQR: 40.0–50.0) for noodles or crackers. Only one serving size was reported on each product label even when the recommended age of use for a product covered a range of ages for infants and children.

**Table 1:** Characteristics of commercially produced complementary foods by type of product

Characteristics	Commercially prepared complementary foods					
	Cereal	Snacks or finger foods	Pureed foods	Mixed meals	Pudding	Other
Recommended age of use (months), % (*n*)						
4+	3 (3)		9 (3)			
6–11+	81 (77)	39 (28)	20 (7)			33 (2)
12+	10 (9)	51 (36)	68 (24)	100 (3)	100 (7)	
24+		4 (3)				17 (1)
No information provided	6 (6)	6 (4)	3 (1)			50 (3)
Serving size on product label (g) median (IQR)	40.0 (25.0–50.0)	21.8 (10.0–40.0)	110.0 (100.0–120.0)	41.0^a^ (25.0–41.0)	b	b
Total, % (*n*)	44 (95)	33 (71)	16 (35)	1 (3)	3 (7)	3 (6)

a Calculation of interquartile range (IQR) for mixed meals not possible because *n* = 3. Minimum and maximum values reported instead.

b No serving size information reported on the labels.

Nearly 70% of CPCF (*n* = 150) reported iron content on the label, but only 58% (*n* = 126) of products were reported to be fortified with iron (Table 2). The 31% (*n* = 67) of CPCF that did not have any information about iron content on the label could not have their iron content calculated including three products that listed iron as a fortificant. Approximately 80% of cereals and snacks or finger food reported iron content in contrast to only 40% of purees. Although 58% (*n* = 126) of all CPCF listed iron in some form as a fortificant, only 19% (*n* = 24) of the iron‐fortified products listed a specific type of iron (e.g. ferrous fumarate). However, all of these products included an iron fortificant recommended by WHO/FAO for effective iron fortification (Allen et al., 2006). Almost all cereal (*n* = 77, 81%) and all mixed meals (*n* = 3, 100%) included added iron, but only about half of snacks or finger food (58%, *n* = 41) and 14% (*n* = 5) of purees were iron fortified. Purees were atypical in that there were 12 products of this type that did not list an iron fortificant, but reported specific iron content on the label, presumably from the product's food components. There were also some snacks or finger food (*n* = 15) that did not list iron as a fortificant but listed iron content as a percent of the RDA. All of these (except one product) listed the iron content as ≤2% of the RDA, so they are consistent with Indonesian labelling law that does not require iron to be listed as an ingredient if the iron content of the food itself is ≤2%.

**Table 2:** Commercially prepared complementary foods for sale by type of product and stratified by report of iron content and iron fortification on the product label

Product type	Iron content given on label			No iron content given on label		Total number of products
	Iron listed as a fortificant		No iron listed as a fortificant, % (*n*)	Iron listed as a fortificant, % (*n*)	No iron listed as a fortificant, % (*n*)	
	Non‐specific iron, % (*n*)	Specific iron fortificant,^a^ % (*n*)				
Cereal	77 (73)	4 (4)	(0)	(0)	19 (18)	95
Snacks or finger foods	37 (26)	21 (15)	21 (15)^b^	(0)	21 (15)	71
Pureed food	(0)	6 (2)	34 (12)	9 (3)^c^	51 (18)	35
Mixed meals	100 (3)	(0)	(0)	(0)	(0)	3
Pudding	(0)	(0)	(0)	(0)	100 (7)	7
Other	(0)	(0)	(0)	(0)	100 (6)	6
Total products	47 (102)	10 (21)	12 (27)	1 (3)	30 (64)	217

a Recommended types of fortified iron include ferrous sulphate, ferrous fumarate, encapsulated ferrous sulphate and ferrous fumarate, electrolytic iron, and ferric pyrophosphate (Allen et al., 2006).

b There were four snacks or finger foods that did not report recommended age of use on the product label so iron content could not be calculated.

c All three of these products listed a specific iron fortificant.

Among CPCF that reported iron content on their labels, 93% (*n* = 139) did so as a percentage of an RDA (Table 3). Close to 60% of those products referenced the Indonesian RDA and another 22% of the products used the RDA from the United States and Canada or United Kingdom (11%, *n* = 16 for each country's RDA). Table 3 shows also the WHO/FAO RNI used for standardized comparison of iron content in this analysis, although these dietary recommendations were never reported on CPCF product labels. Southeast Asian regional RDAs are given also for comparison purposes.

**Table 3:** Comparison of national nutrient recommendations for iron (mg/day) listed on product labels

Country	Recommended iron intake (mg/day) by child age			No. of products with each country‐specific recommended nutrient intake listed on product labels, % (*n*)
	6–11 months	1–3 years	4–6 years	
Indonesia (Indonesia Kementerian Kesehatan RI. Direktorat, 2014)	7^a^	8	9	57 (87)
United States and Canada (Institute of Medicine, 2001)	11	7	10^b^	11 (16)
United Kingdom (British Nutrition Foundation, 2016)	7.8	6.9	6.1	11 (16)
Italy (Società Italiana di Nutrizione Umana, 2014)	11	8	11	2 (3)
Malaysia (National Coordinating Committee on Food and Nutrition, 2017)				1 (2)
15% bioavailability	6	4		
10% bioavailability	9	6		
China (Chinese Nutrition Society, 2014)	10	9		5 (7)
Thailand (Tee & Florentino, 2005)	9.3	5.8		3 (4)
Japan (Japan Dietetic Association, 2015)	5	4.5^c^	5.5^d^	3 (4)
Other^e^				7 (11)
WHO/FAO (WHO/FAO, 2004)				
15% bioavailability	6.2	3.9	4.2	
10% bioavailability	9.3	5.8	6.3	
5% bioavailability	18.6	11.6	12.6	
Southeast Asia (Tee & Florentino, 2005)				
10% bioavailability	9.3	5.8		
7.5% bioavailability	12.4	7.7		
Total				100 (150)

a For 7–11 months.

b For 4–8 years.

c For 1–2 years.

d For 3–5 years.

e Products with iron content data that did not report iron as a percentage of an recommended dietary allowance.

The average calculated iron content of all CPCF was 8.3 (±5.3 *SD*) mg per 100 g of product and 2.6 (±2.0) mg per median serving size. Not surprisingly, unfortified CPCF had much lower iron content per 100 g (0.6 ± 0.6 mg) compared with those fortified with a non‐specific type of iron (8.9 ± 2.6 mg) or a specific iron fortificant (13.6 ± 8.3 mg). However, when iron content per median serving size was examined, the difference in iron content between the two types of iron‐fortified products was not apparent (non‐specific vs. specific fortificant: 3.0 ± 0.9 vs. 3.0 ± 4.1 mg). Close to two thirds (62%, *n* = 15) of CPCF with a specific iron fortificant were snacks or finger food.

Iron content of CPCF per 100 g of product and per median serving size was compared by product type. Cereal (9.6 ± 4.6 mg) and snacks or finger food (8.4 ± 5.4 mg) had the highest concentrations of iron per 100 g. Mixed meals had somewhat less iron per 100 mg (6.4 ± 2.7 mg), and pureed food had less than 1 mg on average (0.8 ± 0.8 mg). However, the larger median serving size of cereal resulted in a larger quantity of fortified iron per median serving size for that product type (cereal: 3.8 ± 1.8 mg) compared with snacks or finger food (1.3 ± 0.9 mg) and mixed meals (2.7 ± 1.1 mg). Pureed food had the lowest iron content (0.9 ± 0.9 mg) because most of these products were not iron fortified.

When comparing iron content to national nutrient recommendations, 100 g of cereal or snacks or finger food provided 105–137% of the Indonesian RDA for iron for children less than 3 years of age (Table 4). The same quantity of mixed meals provided 81–92% of the same RDA, but pureed food contained very little iron. Accordingly, median serving sizes of all CPCF provided a much smaller percentage of the Indonesian RDA for both age groups. A median serving size of cereal provided approximately half of the Indonesian iron RDA whereas a mixed meal product provided one third of the daily requirement and snacks or finger food less than one fifth. The percentage of the Indonesian RDA met by the iron content of CPCF did not vary much whether applied to infants 6–11 months or children 12–36 months because the difference in the recommended daily allowance between the two age categories is 1 mg of iron only (see Table 3).

**Table 4:** Commercially prepared complementary foods for sale by type of product and percentage of Indonesian RDAs and WHO/FAO RNIs for iron met by iron content

Product type	Percentage of Indonesian RDA^a^ for iron met by iron content of a CPCF, mean (95% CI)				Percentage of WHO/FAO RNI^b^ for iron met by iron content of a CPCF, mean (95% CI)				CPCF with iron content data (*n*)
	Per 100 g of product		Per median serving size		Per 100 g of product		Per median serving size		
	RDA for 6–12 months of age	RDA for 12–36 months of age	RDA for 6–12 months of age	RDA for 12–36 months of age	RNI for 6–12 months of age	RNI for 12–36 months of age	RNI for 6–12 months of age	RNI for 12–36 months of age	
Cereal	137 (122–151)	120 (107–133)	55 (49–61)	48 (43–53)	103 (92–114)	165 (147–183)	41 (37–46)	66 (59–73)	77
Snacks or finger foods	120 (99–141)	105 (86–124)	19 (15–22)	16 (13–19)	90 (74–106)	145 (119–171)	14 (11–17)	22 (18–27)	52
Pureed food	11 (5–17)	10 (5–15)	12 (6–19)	11 (5–16)	8 (4–13)	13 (6–20)	9 (4–14)	15 (7–23)	14
Mixed meals	92 (48–137)	81 (42–120)	38 (20–56)	33 (17–49)	69 (36–103)	111 (58–165)	28 (15–42)	46 (24–68)	3

*Note*. CI: confidence interval; CPCF: commercially produced complementary foods; RDA: recommended dietary allowance; RNI: recommended nutrient intakes.

a Indonesia Kementerian Kesehatan RI. Direktorat, 2014.

b WHO/FAO, 2004.

The use of the WHO/FAO RNI for comparison provided a different picture of the contribution of CPCF to daily iron needs of young children, particularly infants 6–11 months, because the iron RNI for that group is much higher (9.3 vs. 5.8 mg iron for infants 6–11 months and children 12–36 months, respectively). One hundred grams of cereal or snacks or finger food met approximately 90–103% of the WHO/FAO recommended iron intake for older infants and 145–165% for children 12–36 months of age. A median serving of cereal provided two thirds of the WHO/FAO RNI for children 12–36 months but only 41% for older infants. Snacks or finger food fared much worse, providing less than a quarter of recommended iron to young children and even less to older infants.

The iron content of snacks or finger food was considered separately for puffs, biscuits or cookies, and noodles or crackers because of the heterogeneity of products of this type (Table 5). Although puffs were more highly fortified with iron than biscuits or cookies (11.9 vs. 6.9 mg per 100 g product), the median serving size of puffs contained about half the iron of biscuits or cookies (0.8 vs. 1.5 mg). This trend was the same for the percentage of the WHO/FAO iron RNI met by the two types of snacks or finger foods. Whereas both puffs and biscuits or cookies appeared to be highly iron fortified per 100 g of product, a median serving of biscuits or cookies met approximately one quarter of the recommended iron intake for young children compared with 14% for a serving of puffs. These products provided an even lower percentage of the recommended iron for infants 6–11 months of age because of the higher iron intakes recommended for that age category. The noodles or crackers in this study did not contain any added iron.

**Table 5:** Types of snacks or finger foods by iron content and percentage of WHO/FAO RNIs for iron met by iron content

Type of snacks or finger foods	Iron content of CPCF (mg), mean (*SD*)		Percentage of WHO/FAO RNIs^a^ for iron met by iron content of a CPCF, mean (95% CI)			
			Per 100 g of product		Per median serving size	
	Per 100 g	Per median serving size	RNI for 6–12 months of age	RNI for 12–36 months of age	RNI for 6–12 months of age	RNI for 12–36 months of age
Puffs (*n* = 16)	11.9 (6.1)	0.8 (0.4)	128 (93–163)	205 (149–261)	9 (7–11)	14 (10–18)
Biscuits or cookies (*n* = 36)	6.9 (4.4)	1.5 (1.0)	74 (58–90)	118 (93–144)	16 (13–20)	26 (20–32)
Noodles or rice crackers (*n* = 15)	0^b^	0	—	—	—	—
Total (*n* = 52)	8.4 (5.4)	1.3 (0.9)	90 (74–107)	145 (119–171)	14 (11–17)	22 (18–27)

*Note*. CI: confidence interval; CPCF: commercially produced complementary foods; RNI: recommended nutrient intakes; SD: standard deviation.

a WHO/FAO, 2004.

b No noodles or rice crackers were iron fortified, and iron content could not be calculated for the four products that had iron content information listed on their labels because recommended age of use was not listed.

## 4 DISCUSSION

In Indonesia, the first foods given to infants include fortified baby foods or other grain‐based porridges (Statistics Indonesia [Badan Pusat Statistik—BPS], National Population and Family Planning Board [BKKBN], and Kementerian Kesehatan [Kemenkes—MOH] & ICF International, 2013). The iron content of the former has not been assessed and home‐made porridges are generally considered to be low in bioavailable iron (Dewey & Brown, 2003; Gibson, Ferguson, & Lehrfeld, 1998; WHO, 1998). This study provides information to assess the quality of iron fortification in CPCF in Indonesia. Few other studies have assessed whether labels provide information on iron content or types of iron used. Masters et al. (2016) reported that of 108 infant cereal product labels from 22 countries reviewed, 53.7% stated iron content and about half contained less iron than reported on the packages.

Nearly half of complementary foods for sale in Bandung, Indonesia, were infant cereals, and about 80% were fortified with iron. Infant cereals have been shown to be an effective means of improving iron status among children 6–12 months of age (Walter et al., 1993; Ziegler, Nelson, & Jeter, 2009), an age at which iron requirements are generally higher than in older children and difficult to meet with unfortified foods (see Table 3). Therefore, ensuring that such products are fortified at optimal levels using appropriate iron forms is important. However, less than 5% of infant cereals in our study reported on the product label the type of fortificant used. The amount of iron provided in infant cereals per 40 g serving was generally appropriate for children 12–36 months of age but fell short of recommended levels (50% of the RDA) for older infants (6–11 months).

One third of all CPCF for sale were snacks or finger food (including biscuits or cookies, puffs, and noodles or crackers), and about 60% were iron fortified. Some have questioned the fortification of snack products and promotion of such products for use by young children because they are often high in added sugar and sodium. Further, they provide only small amounts of iron because the serving size of these products is usually small. Indeed, we found that a serving of snacks or finger food had one third the amount of iron of a serving of infant cereal. Furthermore, there was a significant amount of variability in levels of iron fortification between different types of snacks with puffs having very little iron per serving size compared with biscuits or cookies. Promotion of such complementary snack foods, especially if nutrition claims are given (Sweet et al., 2016), may be detrimental to child nutrition because the fortification may imply the product is an optimal part of the child's diet when it has other negative attributes such as high sodium (as may be found in noodles and crackers) or total sugar (as in puffs, cookies, and biscuits). For example, crackers that were not fortified contain about 25 kcal per serving. However, they are likely to contain sodium that can accustom a child to more salty products.

About one quarter of CPCF sold in Bandung included purees (16% of all products), puddings, mixed meals, and a few other products (teas, oil, and dried chicken) that were either not fortified, fortified at low levels, or scarce. Concern has been expressed about high‐sugar content in purees and puddings, and thus, fortification of these products with iron may not be appropriate also.

We have calculated iron content of CPCF using recommended nutrient intake from various countries per the specification on product labels. Table 3 shows the specific RNI for iron for each country included on the labels in our sample. Assessment of the adequacy of iron content using the Indonesian RDA illustrates a different pattern than using the WHO/FAO RNI because, unlike all other country, regional, and international recommendations shown in Table 3, the Indonesian RDA is lower (7 mg) for infants 7–11 months compared with the RDA for 12–36 months (8 mg). We used both Indonesian RDAs and WHO RNIs, but as Table 3 illustrates, there is a need for harmonization of national and international iron recommendations for young children (National Academies of Sciences, Engineering, and Medicine, 2018). We evaluated the adequacy of iron fortification of CPCF using both the national and international standards to illustrate that iron fortification levels may be inadequate, particularly for infants 6–12 months.

The Indonesian DHS reported that over half of young children consumed iron‐fortified baby foods. Among breastfed children, 59.5% of infants at 6–8 months, 43.8% at 9–11 months, and 23.0% at 12–17 months consumed an iron‐fortified baby food, with similar rates observed among nonbreastfed children (Statistics Indonesia [Badan Pusat Statistik—BPS], National Population and Family Planning Board [BKKBN], and Kementerian Kesehatan [Kemenkes—MOH] & ICF International, 2013). The question used to obtain this information was “Did (NAME) drink/eat any baby food such as Sun, Milna or Cerelac?” Among CPCF in our study, all Cerelac products were infant cereals, whereas Milna and Sun products included infant biscuits and puddings also. All Milna and Sun biscuits were iron fortified, but the puddings were not. This suggests that the DHS could update its questions to determine which types and brands of baby foods are consumed because they are fortified at varied levels and with different forms of iron.

To ensure safe timing of introduction of complementary foods, the WHO's *Guidance to End Inappropriate Promotion of Foods for Infants and Young Children* states that CPCF products must be labelled clearly with an age of introduction of 6 months or above (WHO, 2016). Nearly all (91%) infant cereals identified in this study recommended an age of introduction of at least 6 months. Less than 10% either recommended an age of 4+ months or gave no recommended age. Snacks or finger food (55%) and purees (69%) were more likely to be recommended for use at 12 months of age or older with only 20% (purees) to 39% (snacks or finger foods) recommended starting at 6 months of age instead. It is encouraging to see that most CPCF manufacturers are heeding the WHO's guidance on this issue.

Beal, Tumilowicz, Sutrisna, Izwardy, and Neufeld (2018) reported the importance of early child nutrition to prevent stunting in Indonesia. A study in West Nusa Tenggara Province, Lombok, assessed a community‐based intervention to increase use of fortified biscuits (aimed at the total population, not as complementary foods) among children older than 12 months of age because commercially produced fortified manufactured infant cereal was not typically consumed by such children and because fortified biscuits were locally available (Fahmida et al., 2014). This study found that the median days children consumed fortified biscuits increased from 4 to 7 days after the 6‐month intervention consisting of monthly meetings with mothers where complementary feeding recommendations (including feeding fortified biscuits) and cooking demonstrations were discussed. A further area for research is to assess whether fortified biscuits (and other snacks or finger foods) are appropriate vehicles because their content of sugar, sodium, and transfatty acids may make them inappropriate for young children. A recent workshop proposed that home‐made complementary foods and fortified CPCF are both required to meet the nutrient intakes of infants and young children (GAIN, Savica, Ministry of Health [MOH], 2015).

Semba et al. (2010) and Diana et al. (2017) assessed the relationship of fortified foods (including iron‐fortified milk or formula, fortified noodles, and fortified infant foods) with child growth and/or anaemia status in Indonesia. Semba et al. found no impact of fortified noodles on iron status, and Diana et al. found that infants who consumed fortified foods had higher iron intakes than those who did not, but these intakes represented a small proportion of iron needed in relation to requirements in infants 6 to 12 months of age. Our study shows that understanding the types of foods consumed, their fortification levels, and forms of iron used may help explain such results. The lack of effect on iron status from iron‐fortified CPCF may be explained by low levels of fortification or use of fortificants with low bioavailability.

A strength of this study was the collection of data on a large number of CPCF sold in Bandung through purchases of products in over 40 stores. A limitation of this work was lack of information collected from product labels on energy and other nutrient content, as well as nutrition claims made on product labels. This study would have been strengthened also by the inclusion of laboratory analysis of iron content of CPCF to compare reported and actual iron content. A major concern with CPCF labels reviewed for this study is that often the recommended age of use covered multiple RDA age categories (e.g., 6–24 months) and iron content was reported on the label as a percentage of an iron RDA that differs between 6–11 months and 1–3 years. Only one CPCF manufacturer reported the percentage of the RDA for each specific age group. This created a methodological challenge for our analysis because to compare iron content in products, we needed to calculate the amount of iron based on this combined RDA that may differ from how the manufacturer converted their iron content to a reported percentage of the RDA.

One of the major findings of this study is that even with Indonesian regulations and Codex Alimentarius standards, many products are not labelled appropriately. There is a need for monitoring and enforcement of regulations to ensure optimal product content and labelling. Additionally, more specific guidelines are needed that require labels to report recommended forms of iron, specify a minimum amount of iron fortification per serving, and indicate nutrient levels by child's age because requirements differ. In addition to reporting on percentage of RDA, products should also report iron content per serving and per 100 g as suggested in Codex guidelines. Further research is needed to assess total and free sugar, sodium, energy, transfatty acid and saturated fat content of recommended serving sizes of fortified complementary foods. Such information can help to develop policies to encourage optimal fortified foods and discourage consumption of foods that may lead to increases in nutrition related chronic diseases later in life.

## CONFLICTS OF INTEREST

The authors declare that they have no conflicts of interest.

## CONTRIBUTIONS

MD and SH developed the methodology and analysis plan and prepared the manuscript. MD conducted all analyses. DNH oversaw data collection. MG supervised data collection and management. A collected data on labels and coded them into data collection forms. DI provided guidance on Ministry of Health policy concerns and provided technical advice on study design and execution. All authors reviewed and provided inputs on manuscript drafts.
